# Supplementary material for: Diverse bacteriohemerythrin genes of Methylomonas denitrificans FJG1 provide insight into the survival and activity of methanotrophs in low oxygen ecosystems
Source: mBio. 2025 Sep 25;16(11):e01533-25. doi: 10.1128/mbio.01533-25 (PMC12607906; doi:10.1128/mbio.01533-25)
Supplement: Supplemental material — Supplemental figures, table, and methods. [file mbio.01533-25-s0001.pdf]

Supplemental Materials

Diverse bacteriohemerythrin genes of *Methylomonas denitrificans* FJG1 provide insight into the survival and activity of methanotrophs in low oxygen ecosystems

Cerrise Weiblen[1], K. Dimitri Kits[1], Manuel Kleiner[2], Dominic Sauvageau[3], Lisa Y. Stein[1#]

[1]Department of Biological Sciences, University of Alberta, Edmonton AB, Canada

[2]Department of Plant and Microbial Biology, North Carolina State University, Raleigh, NC, USA

[3]Department of Chemical and Materials Engineering, University of Alberta, Edmonton AB, Canada

# Correspondence: lisa.stein@ualberta.ca

Supplemental Figure S1. Figure 1 with taxon names included.

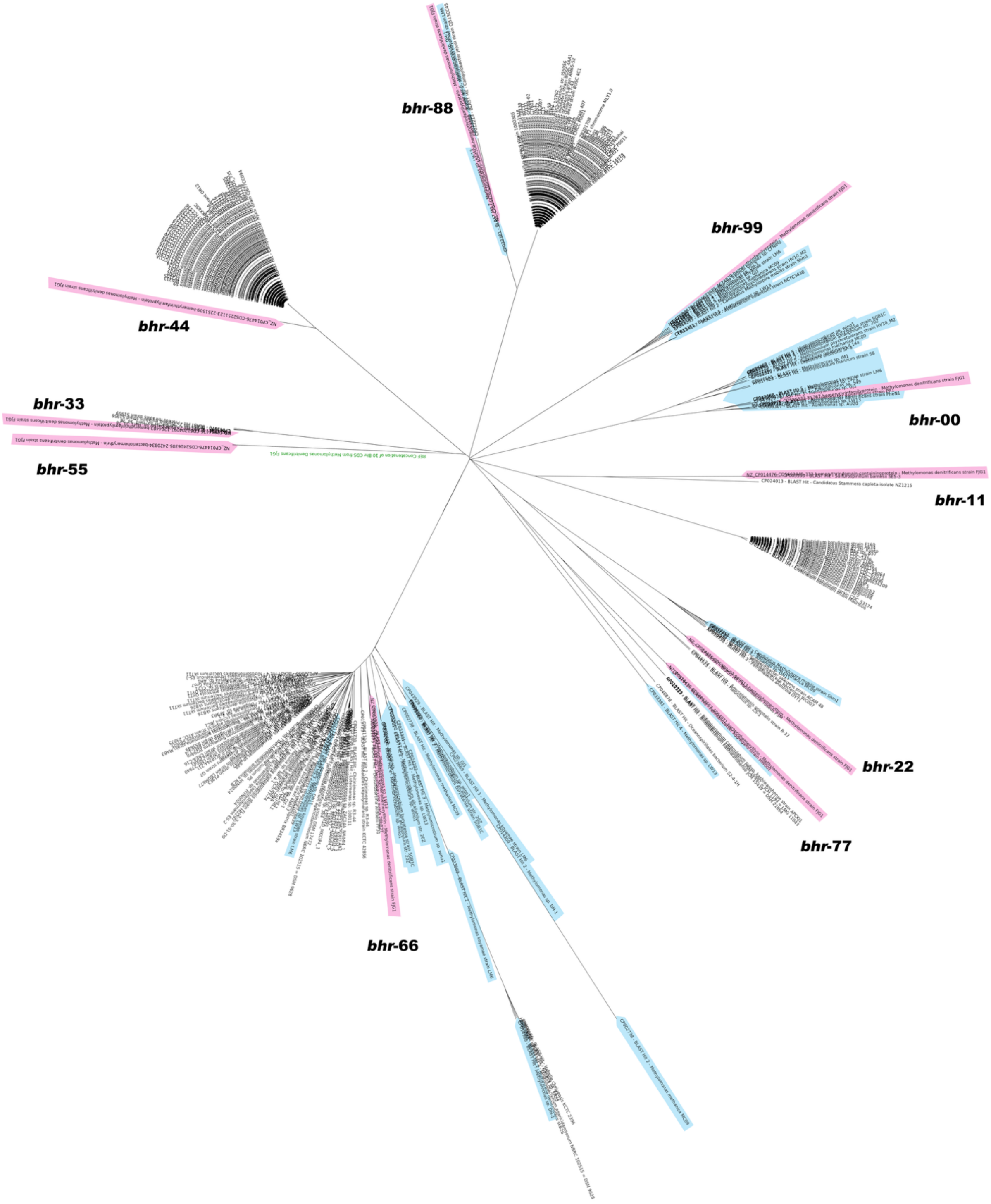

Supplemental Figure S2. Circular alignment of methanotroph genomes created with Blast Ring Image Generator (BRIG). The location of *bhr* genes in *M. denitrificans* FJG1 and other methanotroph genomes are designated by colored bars.

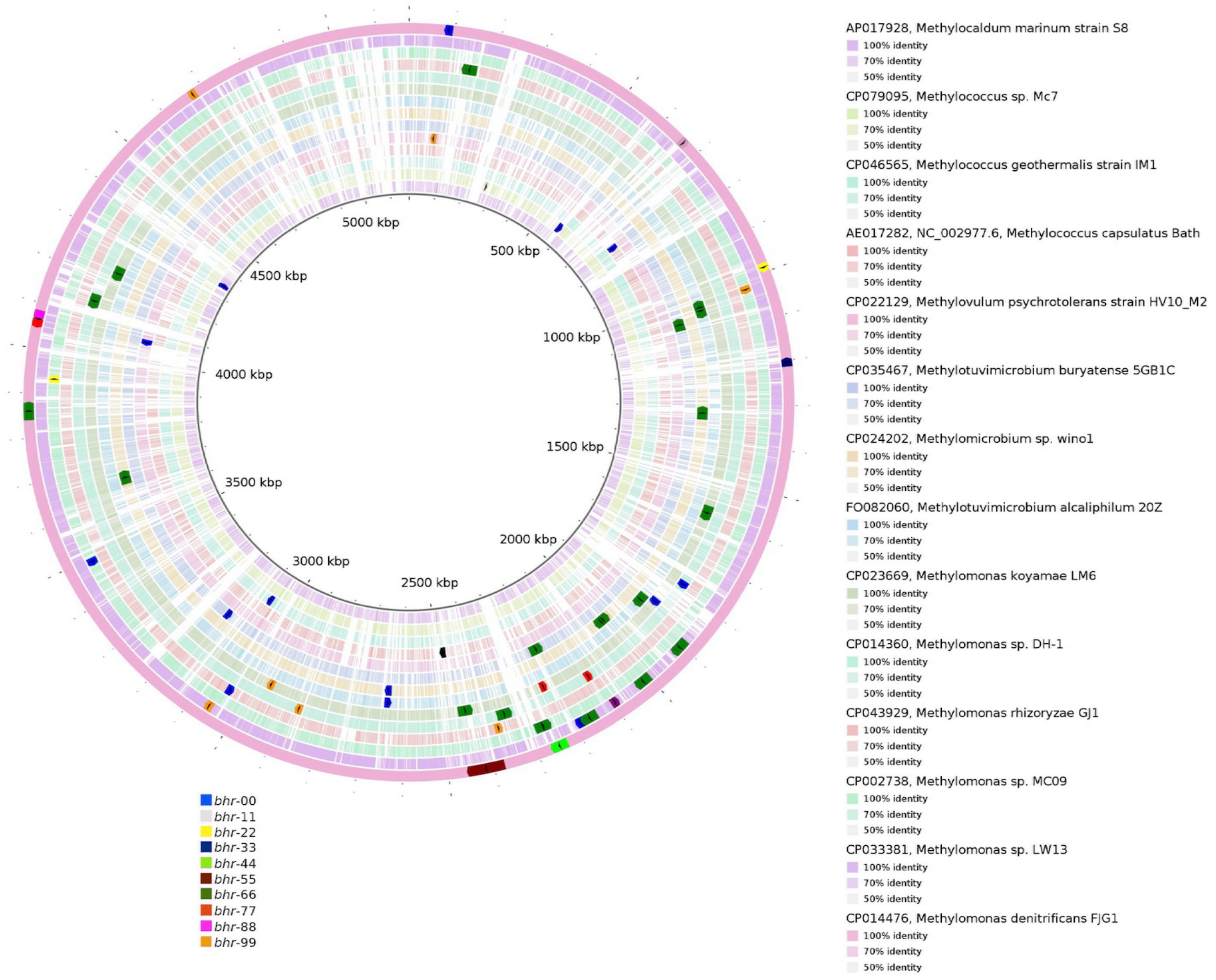

Supplemental Figure S3. mRNA and protein expression levels for *bhr*-22, -33, -44, and -55 and gene neighborhoods for cells experiencing high (24 h) and low (48 h) oxygen levels in *M. denitrificans* FJG1 cultures (Supp. Fig. 5). The levels of mRNA expression (in TPM) for NMS (red/orange/yellow heatmap), protein expression (in normalized NSAF) for NMS (pink/purple heatmap) and mRNA expression (in TPM) for AMS (green/yellow heatmap) for *bhr* and neighboring genes are shown for cells extracted at 24 and 48 h time points from left to right. Gene neighbourhood members are shown on the left in their operon structures and are colour-coded by functional group as per the legend. Up arrows indicate genes encoded on the forward DNA strand, and down arrows indicate genes encoded on the reverse DNA strand.

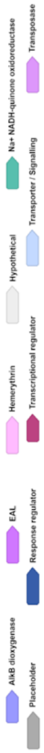

***bhr*-22 neighbourhood**

Gene Locus	Gene Product	NMS TPM			NMS NSAFx10K			AMS TPM		
		24 hr	48 hr	72 hr	24 hr	48 hr	72 hr	24 hr	48 hr	72 hr
JT25_RS04560	EAL domain-containing protein	19	24	17	-	-	-	23	22	17
JT25_RS04565	hypothetical protein	36	58	50	-	-	-	33	39	38
JT25_RS04575	hypothetical protein	139	331	686	-	-	-	202	293	328
JT25_RS04580	thermonuclease family protein	18	30	28	-	-	-	32	29	27
JT25_RS04585	DUF1415 domain-containing protein	11	7	12	-	-	-	7	8	9
JT25_RS04595	IS3 family transposase	18	20	21	-	-	-	40	21	21
JT25_RS04600	bacteriohemerythrin	93	62	45	-	-	-	123	57	47
JT25_RS04605	hypothetical protein	191	154	109	10.96	11.27	10.07	335	157	117
JT25_RS04610	DUF2288 domain-containing protein	123	166	172	-	-	-	134	169	200
JT25_RS04615	DoxX family protein	52	87	98	-	-	-	68	88	83
JT25_RS04620	cupin domain-containing protein	37	57	113	-	-	-	26	63	46
JT25_RS04625	ATP-dependent RNA helicase RhlE	23	15	12	3.15	2.19	2.33	20	19	12
JT25_RS04630	PilZ domain-containing protein	92	71	61	-	-	-	106	82	75
JT25_RS04635	ATP-dependent helicase HepA	46	31	19	2.86	2.22	1.83	43	39	19

***bhr*-44 neighbourhood**

Gene Locus	Gene Product	NMS TPM			NMS NSAFx10K			AMS TPM		
		24 hr	48 hr	72 hr	24 hr	48 hr	72 hr	24 hr	48 hr	72 hr
JT25_RS23895	transposase IS3 family transposase									
JT25_RS23900	transposase IS3 family transposase									
JT25_RS23905	hypothetical protein									
JT25_RS23910	transposase IS5 family transposase									
JT25_RS23910	transposase IS5 family transposase									
JT25_RS25020	hypothetical protein									
JT25_RS10345	hypothetical protein	607	3436	3123	-	-	-	1736	963	634
JT25_RS10350	IS256 family transposase	54	129	139	-	-	-	65	78	80
JT25_RS2469(	hypothetical protein									
JT25_RS25025	hypothetical protein									
JT25_RS10365	hypothetical protein	205	204	91	-	-	-	386	186	99
JT25_RS10370	hypothetical protein	13	5	5	-	-	-	10	7	6
JT25_RS10375	hemerythrin family protein	83	72	68	-	-	-	74	65	57
JT25_RS10385	DUF1508 domain-containing protein	13	16	40	-	-	-	7	8	11
JT25_RS10390	hypothetical protein	11	26	28	-	-	-	9	14	13
JT25_RS10395	PKD domain-containing protein	3	4	4	-	-	-	2	3	3

***bhr*-33 neighbourhood**

Gene Locus	Gene Product	NMS TPM			NMS NSAFx10K			AMS TPM		
		24 hr	48 hr	72 hr	24 hr	48 hr	72 hr	24 hr	48 hr	72 hr
JT25_RS05565	ATP-dependent helicase	25	20	18	-	-	-	19	20	15
JT25_RS05570	hypothetical protein	78	65	36	-	-	-	65	60	38
JT25_RS05575	hypothetical protein	46	54	52	-	-	-	56	61	45
JT25_RS05585	histidine kinase response regulator	59	58	42	1.52	1.98	1.95	72	55	40
JT25_RS05590	hemerythrin family protein	29	41	48	-	-	-	23	39	29
JT25_RS05595	hypothetical protein	46	61	53	-	-	-	30	42	40
JT25_RS05600	hypothetical protein	22	37	38	-	-	-	40	27	26
JT25_RS05605	hypothetical protein	197	154	147	-	-	-	120	147	116
JT25_RS05610	hypothetical protein	6	4	5	-	-	-	7	5	4
JT25_RS05615	hypothetical protein	10	21	18	-	-	-	9	15	15
JT25_RS05620	hypothetical protein	3	3	4	-	-	-	2	4	3
JT25_RS05625	radC DNA repair protein RadC	1	2	2	-	-	-	1	1	1
JT25_RS05630	hypothetical protein	4	6	4	-	-	-	3	5	4
JT25_RS05635	site-specific integrase	7	6	5	-	-	-	5	6	4
JT25_RS05640	hypothetical protein	3	4	3	-	-	-	3	3	2
JT25_RS05645	site-specific integrase	3	6	5	-	-	-	3	4	151
JT25_RS05650	NAD-dependent deacylase	6	9	4	-	-	-	5	6	6

***bhr*-55 neighbourhood**

Gene Locus	Gene Product	NMS TPM			NMS NSAFx10K			AMS TPM		
		24 hr	48 hr	72 hr	24 hr	48 hr	72 hr	24 hr	48 hr	72 hr
JT25_RS11110	IS5 family transposase	19	26	35	-	-	-	16	20	25
JT25_RS11115	hypothetical protein	96	102	128	-	-	-	64	96	124
JT25_RS11120	glycosyltransferase	78	61	46	-	-	-	58	60	40
JT25_RS11125	bacteriohemerythrin	31	47	65	0.79	0.49	0.51	32	36	40
JT25_RS11130	luxR-type helix-turn-helix transcriptional regulator	63	60	79	2.18	2.12	1.97	69	50	65
JT25_RS11135	tyrosine-type recombinase/integrase	37	35	28	-	-	-	33	33	20
JT25_RS11140	Fis family transcriptional regulator	43	64	47	-	-	-	42	47	38
JT25_RS11145	hypothetical protein	30	27	29	-	-	-	104	46	35
JT25_RS11150	hypothetical protein	23	22	27	-	-	-	70	52	29
JT25_RS11155	sodium:proton antiporter NhaD	65	88	105	-	-	-	226	201	131
JT25_RS11160	MarR family transcriptional regulator	25	44	40	-	-	-	168	115	54

***bhr-99* neighbourhood**

Gene Locus	Gene Product	NMS TPM			NMS NSAFx10K			AMS TPM		
		24 hr	48 hr	72 hr	24 hr	48 hr	72 hr	24 hr	48 hr	72 hr
JT25_RS21410	Na(+)-translocating NADH-quinone reductase subunit A	594	587	450	15.76	14.95	17.04	932	811	558
JT25_RS21415	NADH:ubiquinone reductase (Na(+)-transporting) subunit B	1271	813	490	-	-	-	1395	1107	603
JT25_RS21420	Na(+)-translocating NADH-quinone reductase subunit C	1046	903	409	15.03	12.75	13.30	1589	1129	535
JT25_RS21425	NADH:ubiquinone reductase (Na(+)-transporting) subunit D	591	505	335	-	-	-	772	661	400
JT25_RS21430	nqrE, NADH:ubiquinone reductase (Na(+)-transporting) subunit E	711	548	305	-	-	-	762	673	425
JT25_RS21435	nqrF, NADH:ubiquinone reductase (Na(+)-transporting) subunit F	987	1053	590	18.91	17.81	18.30	1315	1252	988
JT25_RS21440	nqrM, (Na+)-NQR maturation	315	203	138	-	-	-	193	194	152
JT25_RS21445	alkB, alpha-ketoglutarate-dependent dioxygenase	90	106	56	-	-	-	89	91	54
JT25_RS21450	hemerythrin family protein	170	173	90	-	-	-	193	182	96
JT25_RS21455	exodeoxyribonuclease VII large subunit	26	41	29	0.74	0.29	0.24	50	41	31
JT25_RS21460	cation transporter	78	96	78	-	-	-	68	81	66
JT25_RS21465	class I SAM-dependent methyltransferase	33	43	18	-	-	-	33	33	23
JT25_RS21475	NUDIX domain-containing protein	81	36	27	-	-	-	58	41	37
JT25_RS21480	WHG domain-containing protein	106	101	105	-	-	-	107	111	104
JT25_RS21485	lplT, lysophospholipid transporter	31	29	15	-	-	-	99	61	28
JT25_RS21490	AMP-binding protein	27	53	55	-	-	-	118	130	95

Supplemental Figure S4. TEM image of a single *M. denitrificans* FJG1 cell grown in NMS media.

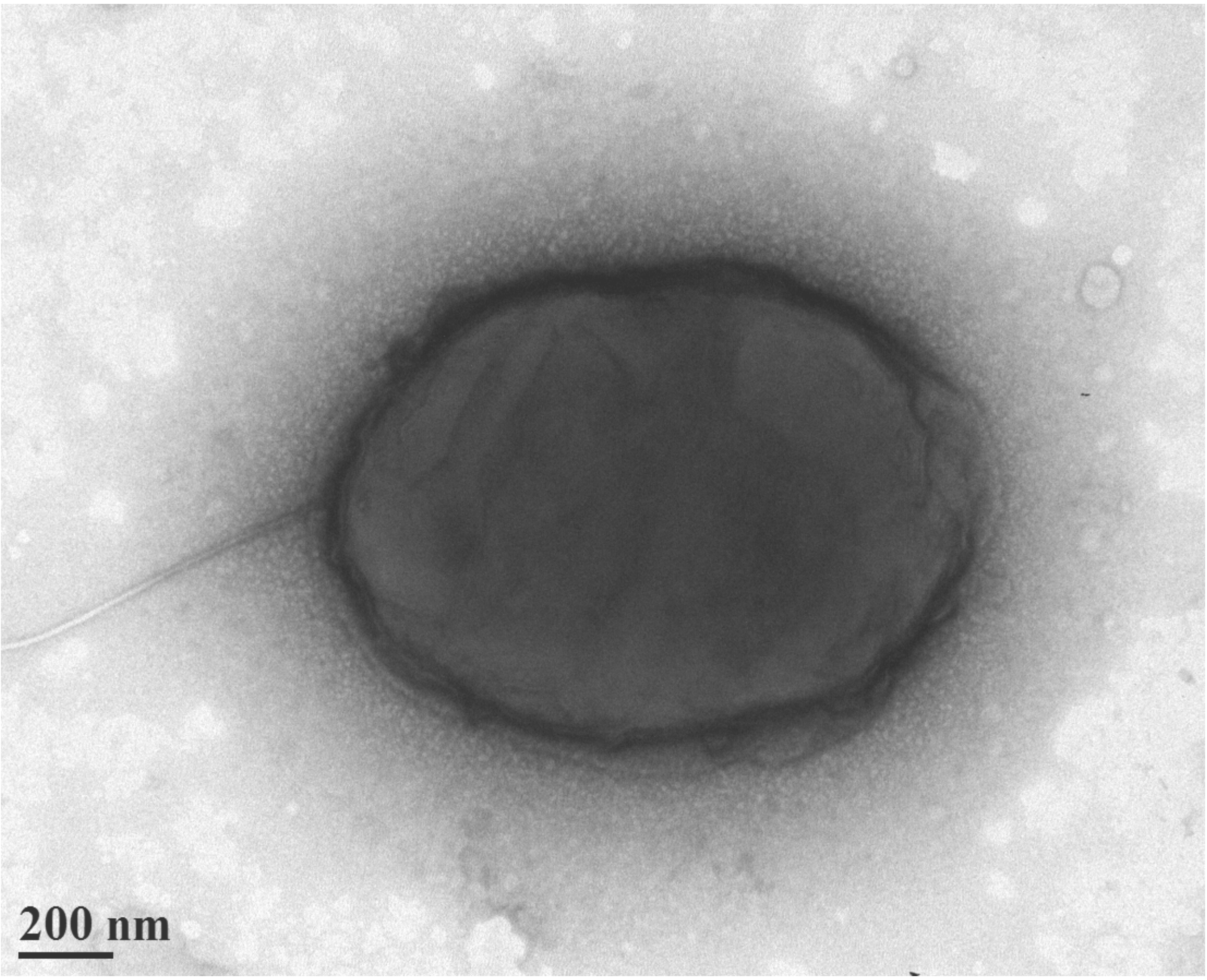

Supplemental Figure S5. Alignment of Bhr-00 protein sequence from *Methylomonas denitrificans* FJG1 with Bhr-Bath protein sequence from *Methylococcus capsulatus* Bath. % amino acid identity is 57.576%.

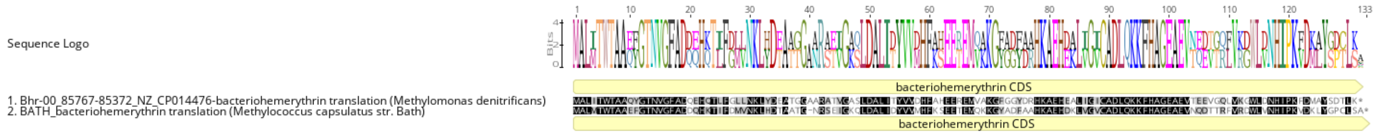

Supplemental Figure S6. Gene neighborhoods of *bhr*-66 of non-methanotroph genomes.

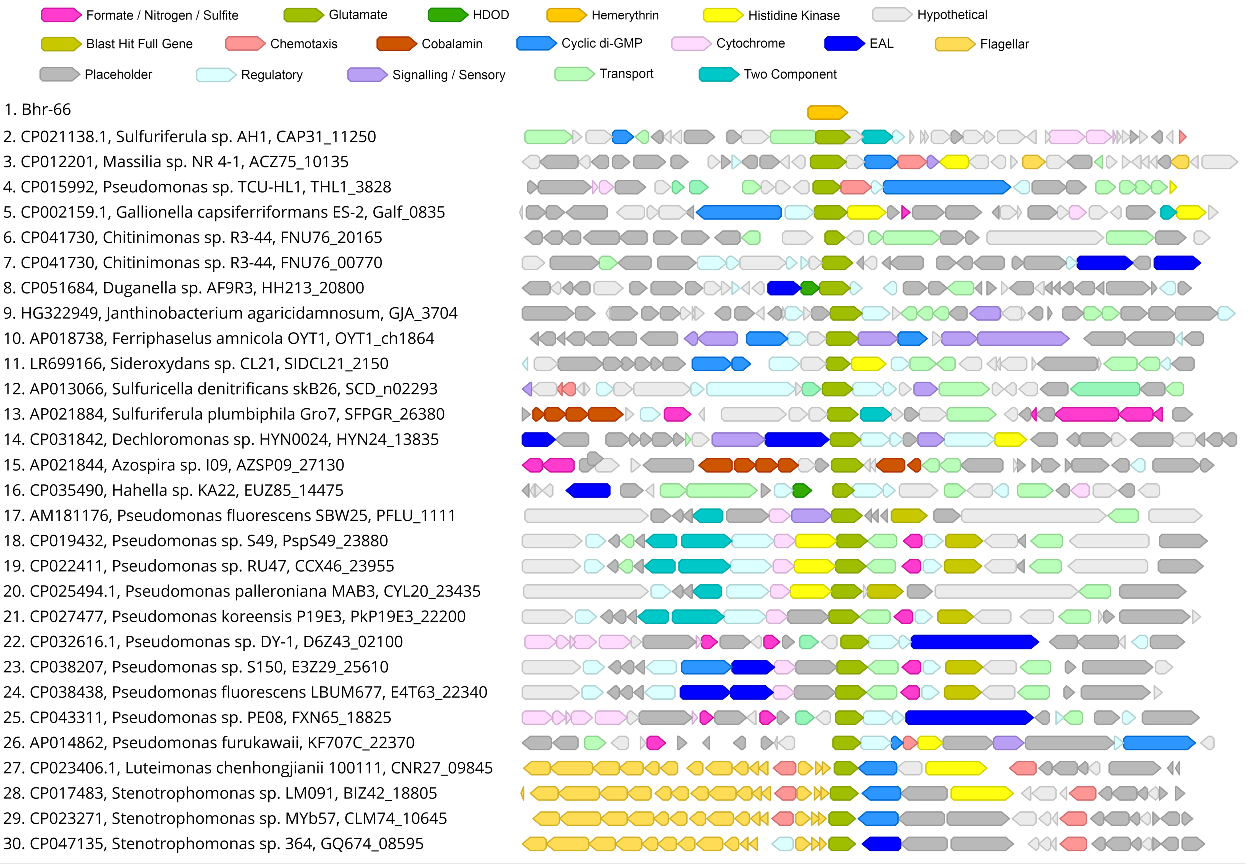

Supplemental Figure S7. Growth curve of *M. denitrificans* FJG1 on NMS or AMS media. (A) Growth over time. (B) Oxygen consumption over time. Reprinted with permission from: Kits KD, Klotz MG, Stein LY. 2015. Methane oxidation coupled to nitrate reduction under hypoxia by the Gammaproteobacterium *Methylomonas denitrificans*, sp. nov. type strain FJG1. Environ Microbiol 17:3219-3232.

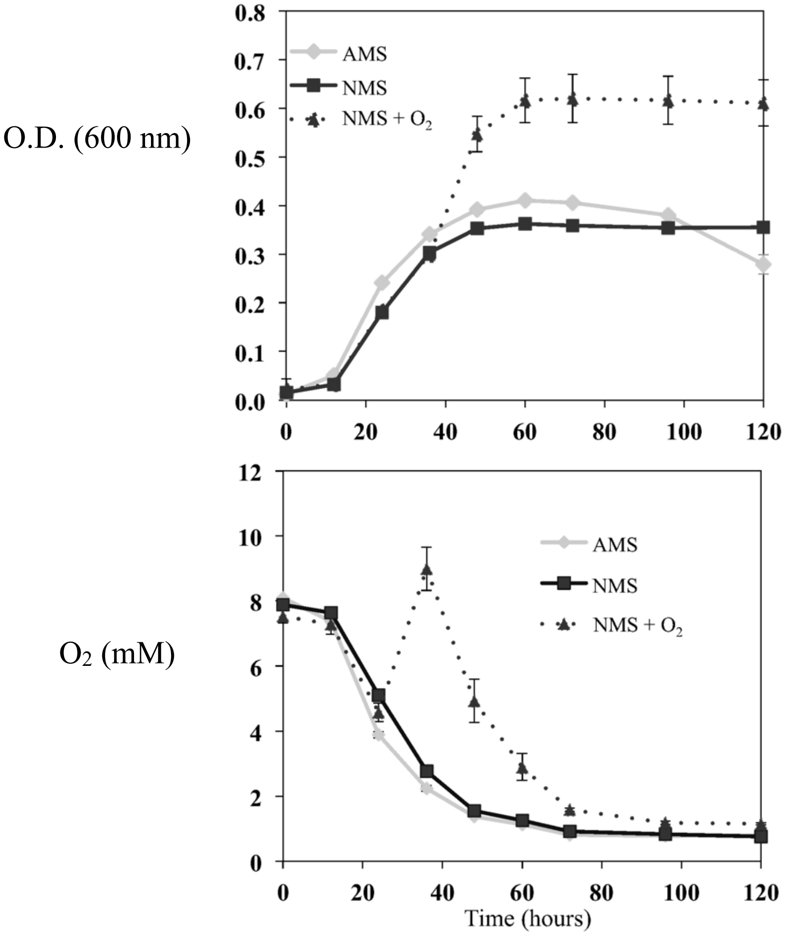

Supplemental Table 1. Differential expression of neighboring genes upstream and downstream of the *bhr*-00, -11, -66, and -77/-88 homologues from *M. denitrificans* FJG1 based on RNAseq data at 24 h versus 48 h of growth in NMS and AMS media.

Gene	Annotation	Locus_tag	bp		Nitrate Treatment (NMS)					Ammonium Treatment (AMS)				
					Raw Expression (TPM)		Differential Expression 24h vs 48h			Raw Expression (TPM)		Differential Expression 24h vs 48h		
					24h	48h	log2 ratio	adjusted p-value	norm log2 ratio	24h	48h	log2 ratio	adjusted p-value	norm log2 ratio
**bhr-00 biotin neighbourhood**														
	twin transmembrane helix small protein	JT25_RS00355	261	F	79.29	34.01	-1.09	0.000	0.89	125.15	56.68	-0.82	0.000	0.48
	cytochrome c oxidase subunit 3	JT25_RS00360	876	R	460.92	282.04	-0.75	0.000	1.23	392.14	388.57	0.16	0.000	1.46
	cytochrome c oxidase assembly protein	JT25_RS00365	543	R	1,941.07	1,120.97	-0.97	0.000	1.01	1,900.11	1,421.06	-0.33	0.000	0.98
ctaD	cytochrome c oxidase subunit I	JT25_RS00370	1617	R	1,103.57	597.90	-1.01	0.000	0.97	875.64	771.26	-0.19	0.000	1.11
coxB	cytochrome c oxidase subunit II	JT25_RS00375	1131	R	1,545.43	1,133.14	-0.60	0.000	1.37	1,458.49	1,556.46	0.07	0.000	1.37
bhr-00	hemerythrin family protein	JT25_RS00380	396	R	4,657.50	13,150.82	1.35	0.000	3.33	7,706.16	28,626.86	1.91	0.000	3.21
bioD	dethiobiotin synthase	JT25_RS00385	696	R	29.97	22.39	-0.38	0.071	1.60	23.62	25.97	-0.10	0.517	1.20
bioC	malonyl-ACP O-methyltransferase BioC	JT25_RS00390	789	R	30.27	24.52	-0.66	0.002	1.32	28.87	21.38	-0.29	0.041	1.01
bioH	pimeloyl-ACP methyl ester esterase BioH	JT25_RS00395	762	R	51.20	38.11	-0.66	0.000	1.32	76.59	36.15	-0.20	0.095	1.10
bioF	8-amino-7-oxononanoate synthase	JT25_RS00400	1161	R	34.95	33.30	-0.24	0.111	1.74	44.40	33.37	-0.13	0.220	1.17

bioB	biotin synthase BioB	JT25_RS00405	990	R	35.14	43.71	-0.07	0.751	1.91	30.27	36.99	0.01	0.910	1.31
**bhr-11 nitrogen neighbourhood**														
narA	nitrate reductase subunit alpha	JT25_RS02985	3735	F	32.81	86.13	1.03	0.000	3.00	35.84	19.24	-0.85	0.000	0.45
narH	nitrate reductase subunit beta	JT25_RS02990	1596	F	42.38	105.88	0.94	0.000	2.91	43.79	25.92	-0.91	0.000	0.39
narJ	nitrate reductase molybdenum cofactor assembly chaperone	JT25_RS02995	720	F	38.92	79.57	0.99	0.000	2.97	32.73	28.02	-0.49	0.000	0.81
narI	respiratory nitrate reductase subunit gamma	JT25_RS03000	678	F	57.48	142.69	1.25	0.000	3.22	59.90	36.01	-0.99	0.000	0.31
	multicopper oxidase Domain-containing protein	JT25_RS03005	1059	F	26.22	69.79	1.16	0.000	3.13	31.77	22.32	-0.23	0.082	1.07
	hypothetical protein	JT25_RS03010	219	F	97.95	199.99	1.74	0.000	3.72	128.30	61.83	-0.63	0.000	0.67
bhr-11	hemerythrin domain-containing protein	JT25_RS03015	333	F	38.22	62.58	1.17	0.000	3.15	75.47	23.55	-1.14	0.000	0.16
	HPP family protein	JT25_RS03020	1185	F	45.08	54.54	0.57	0.000	2.55	33.86	44.21	0.12	0.188	1.42
	NAD(P)/FAD-dependent oxidoreductase	JT25_RS03025	1230	R	51.21	66.18	0.05	0.568	2.03	39.95	36.87	0.43	0.000	1.73
phzF	PhzF family phenazine Biosynthesis protein	JT25_RS03030	921	R	50.59	61.40	0.04	0.707	2.02	20.63	31.09	0.31	0.013	1.61
nirD	nitrite reductase small subunit D	JT25_RS03035	336	R	40.85	50.84	0.41	0.008	2.39	18.39	28.08	0.32	0.133	1.62
nirB	nitrite reductase large subunit B	JT25_RS03040	2541	R	21.28	55.98	0.75	0.000	2.72	14.80	11.33	0.56	0.000	1.86
	formate/nitrite transporter family protein	JT25_RS03045	810	F	22.93	130.56	2.08	0.000	4.06	8.05	5.42	-0.82	0.001	0.48

	bifunctional protein-serine/threonine kinase/phosphatase	JT25_RS03050	1851	F	14.22	46.66	1.34	0.000	3.32	5.07	5.22	-0.24	0.235	1.06
narK	NarK family nitrate/nitrite MFS transporter	JT25_RS03055	1476	F	7.48	10.10	0.91	0.000	2.89	1.86	2.42	0.10	0.772	1.41
**bhr-66 neighbourhood**														
lexA	transcriptional repressor LexA	JT25_RS17770	618	R	6.57	12.02	0.62	0.103	2.60	10.07	8.74	-0.49	0.053	0.81
	response regulator	JT25_RS17775	807	R	21.14	25.57	0.01	0.965	1.99	24.80	22.30	-0.42	0.003	0.88
	DUF447 family protein	JT25_RS17780	564	F	132.49	242.09	0.71	0.000	2.68	155.51	222.25	0.64	0.000	1.94
	hypothetical protein	JT25_RS17785	687	F	100.03	139.33	0.36	0.000	2.34	97.49	123.79	0.06	0.453	1.36
	EAL domain-containing protein	JT25_RS17790	3303	F	52.09	49.09	-0.25	0.000	1.73	66.52	44.21	-0.26	0.000	1.04
	AEC family transporter	JT25_RS17795	924	F	52.51	36.70	-0.56	0.000	1.42	40.93	42.50	-0.22	0.021	1.08
	competence/damage-inducible protein A	JT25_RS17800	1227	F	16.21	18.39	0.09	0.694	2.07	22.13	19.04	0.24	0.091	1.54
	ATP-binding cassette domain-containing protein	JT25_RS17805	1887	F	35.74	43.64	0.03	0.822	2.01	53.44	44.50	-0.19	0.004	1.11
	hypothetical protein	JT25_RS17810	420	R	68.69	73.58	0.09	0.633	2.07	68.77	68.53	-0.25	0.027	1.05
bhr-66	bacteriohemerythrin	JT25_RS17815	1785	F	48.44	63.83	0.28	0.002	2.26	45.44	53.34	0.17	0.013	1.47
	response regulator	JT25_RS17820	1320	F	39.68	45.03	-0.05	0.763	1.93	41.03	38.23	-0.04	0.706	1.26
ppk2	polyphosphate kinase 2	JT25_RS17825	783	F	36.87	50.14	0.49	0.002	2.47	37.17	47.23	0.07	0.542	1.37
	hypothetical protein	JT25_RS17830	645	R	86.78	75.61	-0.24	0.072	1.74	111.16	110.81	0.23	0.003	1.53
	hypothetical protein	JT25_RS17835	2244	R	55.70	67.73	0.19	0.013	2.17	91.41	123.89	0.50	0.000	1.80

	hypothetical protein	JT25_RS17840	1140	R	7.31	17.17	0.83	0.003	2.80	9.25	13.59	0.27	0.113	1.57
	hypothetical protein	JT25_RS17845	561	F	1.96	1.29	-0.23	0.782	1.75	1.00	1.59	0.30	0.596	1.60
gspD	type II secretion system secretin GspD	JT25_RS17850	1908	F	1.38	4.89	0.83	0.230	2.81	0.85	1.14	0.18	0.682	1.48
gspE	type II secretion system ATPase GspE	JT25_RS17855	1512	F	1.27	2.08	0.21	0.743	2.19	0.95	1.57	0.35	0.409	1.66

bhr-77, bhr-88 chemotaxis neighbourhood

	chemotaxis response regulator protein-glutamate methylesterase	JT25_RS18665	1095	R	44.02	103.47	1.08	0.000	3.06	46.36	64.11	0.54	0.000	1.84
cheD	chemotaxis protein CheD	JT25_RS18670	597	R	34.68	163.80	1.96	0.000	3.94	70.61	80.00	0.33	0.001	1.63
cheR	Protein-glutamate O-methyltransferase	JT25_RS18675	807	R	58.08	199.36	1.55	0.000	3.53	53.85	101.73	0.65	0.000	1.95
	hypothetical protein	JT25_RS18680	1209	F	186.80	602.19	1.59	0.000	3.57	196.48	406.79	0.92	0.000	2.22
	response regulator	JT25_RS18685	369	F	312.41	1,079.75	1.64	0.000	3.62	417.07	749.70	1.04	0.000	2.34
	STAS domain-containing protein	JT25_RS18690	333	F	214.74	532.60	1.21	0.000	3.19	238.96	386.06	0.87	0.000	2.17
cheA	chemotaxis protein CheA	JT25_RS18695	2202	F	127.43	297.19	1.08	0.000	3.06	111.08	191.32	0.52	0.000	1.82
	HAMP domain-containing protein	JT25_RS18700	2034	F	176.46	453.17	1.21	0.000	3.19	200.61	324.35	0.71	0.000	2.02
cheW	purine-binding chemotaxis protein CheW	JT25_RS18705	546	F	209.45	446.22	0.99	0.000	2.97	297.34	336.69	0.41	0.000	1.71
bhr-77	bacteriohemerythrin	JT25_RS18710	405	R	107.16	176.26	0.62	0.000	2.60	146.80	119.38	0.07	0.470	1.37
bhr-88	hemerythrin family protein	JT25_RS18715	414	R	50.66	80.73	0.59	0.000	2.57	64.90	73.10	0.22	0.071	1.52

	methyl-accepting chemotaxis protein	JT25_RS18720	1878	R	21.25	39.93	0.51	0.001	2.49	24.06	33.64	0.23	0.007	1.53
	formate--tetrahydrofolate ligase	JT25_RS18725	1674	R	61.58	69.40	0.13	0.181	2.11	82.37	74.66	-0.29	0.000	1.01

Normalization

rpoB	DNA-directed RNA polymerase Subunit beta	JT25_RS14695	4077	F	418.02	113.34	-1.98	0.000		421.34	162.99	-1.30	0.000	

Supplemental Methods:

The RNAseq and proteomics experiments were performed in 2015 by Kerim Kits. A portion of the RNAseq data was analyzed and published by Kits et al. (16) comprising the 24 h and 48 h time points of a six-point time course (24, 36, 48, 60, 72, 96, and 120 h). In that study, the data were calculated and reported using R and expression values were reported in RPKM (16). The proteome experiments were generated at the same time as the RNAseq experiments and published in the PhD thesis of D. Kits. The raw Illumina data from the RNAseq experiment were reanalyzed for the present study using DESeq with values reported in TPM. Only the 24, 48 and 72 h time points were used to streamline the findings while still reporting temporal changes in transcript and protein expression levels. The proteome data has been formally reported here to connect protein expression with matching RNAseq data for the *bhr* homologs and their neighboring genes across the same time points (Figs. 5 and S3).
